# Post-resuscitation pneumothorax: retrospective analysis of incidence, risk factors and outcome-relevance

**DOI:** 10.1186/s13049-024-01260-8

**Published:** 2024-09-05

**Authors:** Daniel Auinger, David Hötzer, Paul Zajic, Simon Orlob, Stefan Heschl, Stephanie Fida, Philipp Zoidl, Gabriel Honnef, Herwig Friedl, Freyja-Maria Smolle-Jüttner, Gerhard Prause

**Affiliations:** 1 Division of Anaesthesiology and Intensive Care Medicine 1, Department of Anaesthesiology and Intensive Care Medicine, Medical University of Graz, Graz, Styria Austria; 2 Division of Anaesthesiology and Intensive Care Medicine 2, Department of Anaesthesiology and Intensive Care Medicine, Medical University of Graz, Graz, Styria Austria; 3 Institute of Statistics, Graz University of Technology, Graz, Styria Austria; 4 Division of Thoracic and Hyperbaric Surgery, Department of Surgery, Medical University of Graz, Graz, Styria Austria

**Keywords:** Cardiopulmonary resuscitation, Pneumothorax, Out-of-hospital cardiac arrest, CPR-related injuries

## Abstract

**Background:**

Pneumothorax may occur as a complication of cardiopulmonary resuscitation (CPR) and could pose a potentially life-threatening condition. In this study we sought to investigate the incidence of pneumothorax following CPR for out-of-hospital cardiac arrest (OHCA), identify possible risk factors, and elucidate its association with outcomes.

**Methods:**

This study was a retrospective data analysis of patients hospitalized following CPR for OHCA. We included cases from 1st March 2014 to 31st December 2021 which were attended by teams of the physician staffed ambulance based at the University Medical Centre Graz, Austria. Chest imaging after CPR was reviewed to assess whether pneumothorax was present or not. Logistic regression analysis was performed to identify factors for the development of pneumothorax relevant and to assess its association with outcomes [survival to hospital discharge and cerebral performance category (CPC)].

**Results:**

Pneumothorax following CPR was found in 26 out of 237 included cases (11.0%). History of obstructive lung disease was significantly associated with presence of pneumothorax after CPR. This subgroup of patients (n = 61) showed a pneumothorax rate of 23.0%. Pneumothorax was not identified as a relevant factor to predict survival to hospital discharge or favourable neurological outcome (CPC1 + 2).

**Conclusions:**

Pneumothorax may be present in greater than one in ten patients hospitalized after CPR for OHCA. Pre-existent obstructive pulmonary disease seems to be a relevant risk factor for development of post-CPR pneumothorax. ClinicalTrials.gov ID: NCT06182007 (retrospectively registered).

*Trial Registration*: NCT06182007 (retrospectively registered)

## Background

Out-of-hospital cardiac arrest has an annual incidence of 67–170 per 100,000 inhabitants in Europe [1]. High quality chest compressions are considered a key element of effective cardiopulmonary resuscitation [2]. Traumatic injuries may occur as negative side-effects of CPR. Type and prevalence of CPR-associated injuries have been described in several studies [3–6]. Some of them are potentially life-threatening including pneumothorax, an entry of air into the pleural space.

Tension pneumothorax is present when there is a one-way valve mechanism that allows air to enter the pleural space with every breath but does not allow for its escape. The trapped air builds up an increased pleural pressure which results in a life-threatening condition characterized by both respiratory and hemodynamic compromise [7]. Positive-pressure ventilation can potentially convert pneumothorax into a tension pneumothorax [8].

Diagnosis of pneumothorax is most commonly achieved by chest X-ray (CXR) in hospital[9]. Alternative methods are lung ultrasound and computed tomography (CT), the latter is considered to be the gold standard in pneumothorax detection [10]. Lung ultrasound has also been reported to have superior sensitivity compared to CXR [11] and is suitable for rapid imaging bedside or in the prehospital setting [12]. Rate of pneumothorax following CPR varies widely in literature, ranging from 2.5% up to 26%[3–6, 13, 14]. To the best of our knowledge, factors contributing to the development of post-CPR pneumothorax or its potential impact on outcomes have not been studied exhaustively.

In the European Resuscitation Council (ERC) guidelines 2021 tension pneumothorax is listed among the 4 H’s and 4 T’s, the potentially reversible causes of cardiac arrest [15]. Rapid diagnosis and treatment either by needle decompression or thoracostomy are crucial [8].

In this study, we seek to investigate the incidence of pneumothorax following cardiopulmonary resuscitation for out-of-hospital cardiac arrest, identify possible risk factors, and elucidate its association with outcomes.

## Methods

### Study design and setting

This was a retrospective analysis of routine data, collected by a single emergency medical service (EMS) system. The study site was Graz, Austria, where there is a tiered EMS system. For emergencies perceived as potentially life-threatening both a paramedic-staffed and a physician-staffed vehicle respond [16]. There are 2 ground-based physician-staffed ambulance systems serving the city of Graz and its suburban area with a cumulative population of approximately 453,000 [17]. During daytime there is also a physician-staffed rescue helicopter available, which responds to primary and secondary missions in the surroundings of Graz and the eastern part of the province Styria.

OHCA cases, to which the physician response unit located at the University Medical Centre Graz responded, were included in this study. Advanced Life Support (ALS) was provided according to the latest ERC guidelines. Patients were transported to one of the two receiving hospitals in Graz responsible for cardiac arrest patients. Post-resuscitation care was administered in accordance to the latest version of the applicable guidelines [18]. Whenever pneumothorax was diagnosed, the surgeon on call was consulted.

This study was approved by the Ethics Committee of the Medical University of Graz (IRB00002556, decision number 28-168 ex 15/16) before commencement of the study. The need for informed consent was waived as data was retrieved retrospectively in a pseudonymized fashion. All methods and analyses performed adhered to the applicable STROBE guideline [19].

### Selection of participants

Adult patients (≥ 18 years) hospitalised after OHCA who received chest compressions and underwent chest imaging within the first 12 h after cardiac arrest were eligible for this study. Exclusion criteria were traumatic cardiac arrest, chest trauma/pneumothorax/thoracic surgery within 1 month prior to cardiac arrest and cases with insufficient data quality.

### Measurements

Routine clinical data was collected using an electronic documentation system and database (MEDEA, iLogs, Klagenfurt, Austria). The dataset is based on the minimal data set in German emergency medicine (MIND3), a standard for documentation in prehospital physician response systems in German-speaking countries defined and authorized by the German Interdisciplinary Society of Intensive Care and Emergency Medicine (DIVI) [20]. Eligible patients from March 2014 until December 2021 were identified in the database. Further data was retrieved from the hospital information system.

### Outcomes

The presence or absence of pneumothorax was derived from the radiology report of the first chest imaging procedure performed after hospital admission and, if prehospital lung ultrasound was performed, additionally from the emergency physician protocol. When pneumothorax was present, we also reviewed whether insertion of a chest tube was performed or not.

Variables and outcomes ascertained are built up of the core elements of the latest version of the Utstein Resuscitation Registry Template for OHCA[21]. This includes sex, age, etiology of arrest, response times of the first emergency medical service team and emergency physician, witnessed cardiac arrest, bystander CPR/AED (automated external defibrillator), arrest location, first monitored electrocardiogram (ECG) rhythm, defibrillation time, drugs given, reperfusion attempted and target temperature management. Additional variables studied were pre-existing health status represented by the Pre Emergency Status Assessment (PESA) [22], history of lung disease, no-flow-time (time from collapse until initiation of CPR), use of a mechanical chest compression device and prehospital CPR duration.

The primary outcome was incidence of pneumothorax, secondary outcomes were survival to hospital discharge and favourable neurological condition at hospital discharge defined by a cerebral performance category (CPC) of 1 or 2[23].

### Analysis

Statistical analysis was performed with R-4.2.2, a free software environment for statistical computing and graphics [24]. The set of predictors for the logistic regression models was chosen based on a preliminary exploratory analysis and on recommendations from literature. This approach ensured that there was no bias due to the omission of relevant predictors and that variance of the estimates did not increase due to many irrelevant terms in the model [25]. Changes in the respective adjusted odds ratios caused either by increasing the size of a predictor by one unit or by changing its category were also examined. An effect was considered as statistically significant if the two-sided 95% confidence interval of the odds ratio did not cover the value of one.

The area under the receiver operating characteristic (ROC) curve was estimated to assess how correctly a logistic regression model classifies. For this purpose, the available data were randomly divided into a training (70%) and a test (30%) dataset. Since this classification result depends strongly on the data split, this random partitioning was repeated 1000 times and the median and quantiles of all Monte Carlo replicates of the area under the curve (AUC) were calculated and reported.

## Results

### Characteristics of study subjects

**Fig. 1:**
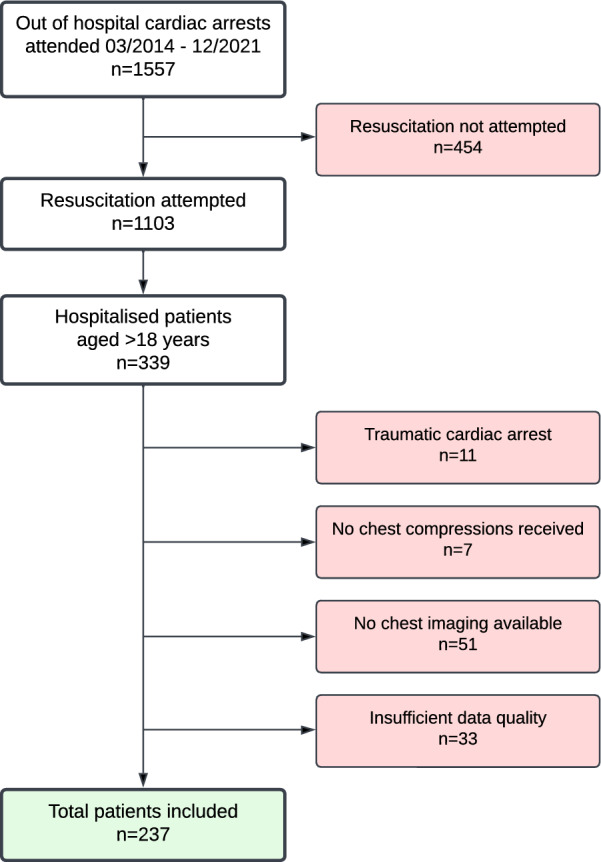
Patient selection flow chart

Of the 339 patients hospitalized after OHCA, 237 patients were included in our study. The selection process is depicted in Fig. 1. Mean age was 65.3 years (SD 15.6) in a range of 18–95 years. 172 patients (72.6%) were men. 182 persons (76.8%) experienced witnessed cardiac arrest, 140 by layperson, 42 by EMS. 127 persons (53.6%) received bystander CPR by layperson. Shockable initial rhythm could be observed in 93 cases (39.2%). For further details please see Table 1.

**Table 1:** Patient, process and outcome characteristics of the entire study population and the subgroups “no pneumothorax” and “pneumothorax”

	All patients		No pneumothorax		Pneumothorax	
	237	100%	211	89.0%	26	11.0%
Patient						
Male gender	172	72.6%	158	74.9%	14	53.8%
Age (mean ± SD [years])	65.3	15.6	64.6	15.6	68.6	15.2
PESA						
1	25	10.5%	22	10.4%	3	11.5%
2	80	33.8%	73	34.6%	7	26.9%
3	111	46.8%	98	46.4%	13	50.0%
4	21	8.9%	18	8.5%	3	11.5%
Etiology						
Medical	172	72.6%	154	73.0%	18	69.2%
Drug overdose	4	1.7%	4	1.9%	0	0.0%
Drowning	1	0.4%	0	0.0%	1	3.8%
Asphyxia	35	14.8%	30	14.2%	5	19.2%
Not recorded	25	10.5%	23	10.9%	2	7.7%
History of lung disease						
None	162	68.4%	150	71.1%	12	46.2%
Obstructive	61	25.7%	47	22.3%	14	53.8%
Inflammatory/Infectious	9	3.8%	9	4.3%	0	0.0%
Interstitial	1	0.4%	1	0.5%	0	0.0%
Neoplasm	4	1.7%	4	1.9%	0	0.0%
Witnessed cardiac arrest						
Bystander witnessed	140	59.1%	121	57.3%	19	73.1%
EMS witnessed	42	17.7%	38	18.0%	4	15.4%
Unwitnessed	49	20.7%	47	22.3%	2	7.7%
Not recorded	6	2.5%	5	2.4%	1	3.8%
Bystander CPR	127	53.6%	113	53.6%	14	53.8%
Bystander AED						
Used, shock delivered	21	8.9%	20	9.5%	1	3.8%
Used, no shock delivered	6	2.5%	6	2.8%	0	0.0%
Not used	210	88.6%	185	87.7%	25	96.2%
Location						
Home	139	58.6%	127	60.2%	12	46.2%
Work	6	2.5%	6	2.8%	0	0.0%
Recreation/Sports	5	2.1%	5	2.4%	0	0.0%
Public	53	22.4%	44	20.9%	9	34.6%
Nursing	17	7.2%	15	7.1%	2	7.7%
Other	16	6.8%	13	6.2%	3	11.5%
Not recorded	1	0.4%	1	0.4%	0	0.0%
Initial ECG rhythm						
VF	88	37.1%	82	38.9%	6	23.1%
VT	5	2.1%	5	2.4%	0	0.0%
PEA	66	27.8%	55	26.1%	11	42.3%
Asystole	61	25.7%	55	26.1%	6	23.1%
Not recorded	17	7.2%	14	6.6%	3	11.5%
Process						
Response time EMS (mean ± SD [mins])	8.8	4.2	8.9	4.3	8.3	3.1
Response time PRU (mean ± SD [mins])	11.3	5.1	11.4	5.3	10.2	3.5
Defibrillation time (if applicable; mean ± SD [mins])	8.8	7.1	8.9	7.3	8.4	3.2
No-flow-time (mean ± SD [min])	2.8	4.98	2.8	5.12	1.9	3.7
Unknown no-flow-time	30	12.7%	28	13.3%	2	7.7%
Prehospital CPR duration (mean ± SD [mins])	21.8	18.2	22.3	18.8	17.3	10.5
Mechanical CPR	4	1.7%	4	1.9%	0	0.0%
Drugs given						
Adrenaline	180	75.9%	157	74.4%	23	88.5%
Amiodarone	56	23.6%	53	25.1%	3	11.5%
Reperfusion attempted	132	55.7%	120	56.9%	12	46.2%
TTM	113	47.7%	96	45.5%	17	65.4%
Chest imaging						
X-Ray	125	52.7%	116	55.0%	9	34.6%
CT	50	21.1%	38	18.0%	12	46.2%
Lung ultrasound	62	26.2%	57	27.0%	5	19.2%
Chest tube insertion					17	65.4%
Outcome						
Survived Event	201	84.8%	176	83.4%	25	96.2%
Any ROSC	230	97.0%	204	96.7%	26	100.0%
Survival to hospital discharge	90	38.0%	83	39.3%	7	26.9%
CPC at hospital discharge						
1	60	25.3%	54	25.6%	6	23.1%
2	15	6.3%	14	6.6%	1	3.8%
3	9	3.8%	9	4.2%	0	0.0%
4	6	2.5%	6	2.8%	0	0.0%
5	147	62.0%	128	60.7%	19	73.1%

SD, standard deviation; PESA, pre emergency status assessment; EMS, emergency medical service; PRU, physician response unit; CPR, cardiopulmonary resuscitation; AED, automated external defibrillator; ECG, electrocardiogram; VF, ventricular fibrillation; VT, ventricular tachycardia; PEA, pulseless electrical activity; mins, minutes; TTM, target temperature management; CT, computed tomography; ROSC, return of spontaneous circulation; CPC, cerebral performance category

### Pneumothorax

Pneumothorax was present upon hospital admission in 26 out of 237 (11.0%) hospitalized OHCA patients. In 17 (65.4%) cases a chest tube was inserted. The most frequently used initial chest imaging method was CXR (n = 125, 52.7%), followed by lung ultrasound (n = 62, 26.2%) and CT (n = 50, 21.1%). Pneumothorax rates in the subgroups CXR, CT and lung ultrasound were 7.2%, 24.0% and 8.1%, respectively.

**Fig. 2:**
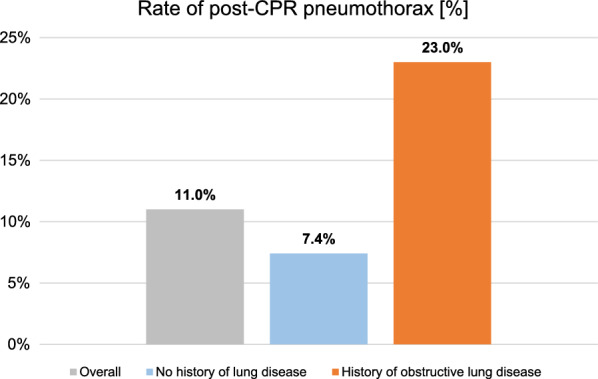
Pneumothorax rates in the overall cohort and the subgroups “no history of lung disease” and “history of obstructive lung disease”

Logistic regression model found history of obstructive lung disease to be significantly associated with the presence of pneumothorax after CPR (OR 3.723, 95% CI 1.611–8.606). Pneumothorax rates in the cohorts “overall”, “no history of lung disease” and “history of obstructive lung disease” were 11,0%, 7.4% and 23.0%, respectively. Please see this finding depicted in Fig. 2. This statistical model had an estimated AUC of 0.671 (95% CI 0.508–0.809).

### Survival to hospital discharge

90 (38.0%) out of 237 patients survived to hospital discharge. Higher age (OR 0.968; 95% CI 0.942–0.996), higher PESA category (PESA3 vs. PESA1: OR 0.218, 95% CI 0.052–0.909; PESA4 vs. PESA1: OR 0.065, 95% CI 0.008–0.551), longer CPR duration (OR 0.921; 95% CI 0.891–0.952) and longer no-flow-time (OR 0.784; 95% CI 0.689–0.891) were identified as statistically significant factors predicting lower probability of survival. Based on this model, the odds to survive drop by 7.9% as a result of 1 min longer CPR duration and by 21.6% when increasing the no-flow-time by 1 min. Non-shockable initial ECG rhythm had significantly smaller odds for survival compared to shockable initial ECG rhythm (OR 4.796; 95% CI 2.098–10.961). Pneumothorax was not found to be a relevant factor in our model (OR 0.579; 95% CI 0.188–1.781). Assessing the predictive performance of this model showed an AUC of 0.849 (95% CI 0.755–0.923).

### Neurological outcome at hospital discharge

75 (31.6%) out of 237 patients had favourable neurological outcome at hospital discharge defined by a CPC of 1 or 2. Higher age (OR 0.955; 95% CI 0.923–0.988), higher PESA category (PESA3 vs. PESA1: OR 0.058, 95% CI 0.010–0.344; PESA4 vs. PESA1: OR 0.039, 95% CI 0.003–0.434), non-shockable initial ECG rhythm (OR 12.75, 95% CI 4.211–38.62), longer CPR duration (OR 0.863, CI 0.813–0.916) and longer no-flow-time (OR 0.629; 95% CI 0.499–0.792) were statistically significant factors predicting lower probability of favourable neurological condition at hospital discharge. Odds of survival with favourable neurological condition were estimated to decrease by 13.7% with each additional minute of CPR duration and by 37.0% with each additional minute of no-flow-time. Again, pneumothorax was not identified as a relevant factor (OR 1.183; 95% CI 0.325–4.303). AUC for this model was estimated with 0.893 (95% CI 0.801–0.954).

## Discussion

In this retrospective study of patients admitted to hospital after out-of-hospital cardiac arrest we found pneumothorax to be present in 11.0% of patients. This rate appears to be in the middle of the wide range of pneumothorax rates following CPR reported in previous studies (2.5–26.4%) [3–6, 13, 14]. Literature in which computed tomography was used for pneumothorax diagnosis found higher rates compared to studies where different diagnostic methods (CXR, CT, lung ultrasound, autopsy) were combined [3–6, 13, 14]. Also in our study the CT-subpopulation showed a higher rate compared to the entire collective (24.0% vs. 11.0%). Computed tomography is known to be the most sensitive method for detection of pneumothorax which could explain this finding [10]. Based on this, it appears that CXR and lung ultrasound may overlook a considerable number of pneumothoraces. However, there is also the question whether a pneumothorax missed by X-ray and lung ultrasound is of clinically significant size and relevance.

History of lung disease was found to be associated with the occurrence of post-CPR pneumothorax. In the subgroup of obstructive lung diseases this association was found to be even stronger and the rate of pneumothorax to be more than doubled compared to the overall cohort. Several pulmonary conditions are known to be a risk factor for development of spontaneous pneumothorax, with chronic pulmonary obstructive disease (COPD) being the most common [7]. It seems that obstructive pulmonary disease makes the lungs less resilient against the mechanical stress of cardiopulmonary resuscitation. Reflecting our findings, we recommend the use of lung ultrasound for pneumothorax detection during and after CPR, especially in the high-risk collective of patients with pre-existent obstructive lung disease. The procedure is quick and relatively easy to perform, can be done on-site and has a higher diagnostic accuracy compared to supine CXR [11, 12].

Evidence of the association of CPR-related injuries and the duration of cardiopulmonary resuscitation in the literature is conflicting. Some authors reported of a significant association of increasing CPR duration with higher rates rib fractures [6, 26] and injuries in general [27]. Others were not able to show a relationship between CPR length and frequency of injuries [4, 28]. Similarly, our study also showed no association between the occurrence of post-CPR pneumothorax and CPR duration.

Younger age, lower PESA category, shorter CPR duration, shorter no-flow-time and shockable initial ECG rhythm were found to be predictive for higher probability of both survival to hospital discharge and favourable neurological outcome (defined as CPC of 1 or 2). This corresponds well with known predictors and scoring systems for CPR outcome from literature [29–32]. We observed higher rates of both survival to hospital discharge and favourable neurological outcome in the no-pneumothorax subgroup compared to the pneumothorax subgroup (25.9% vs. 39.5% and 25.9% vs. 32.1%; respectively). Multivariable logistic regression analysis, however, failed to identify pneumothorax as an independent risk factor for worsened outcome.

Adrie et al. [29], Amacher et al. [30], Maupain et al. [31] and Sasson et al. [32] our statistical models showed the dramatic impact of longer no-flow-time on probability of survival to hospital discharge (increase of 1 min = 21.7% lower chance for survival to hospital discharge) and favourable neurological outcome (increase of 1 min = 37.0% lower chance to have CPC of 1 or 2). No-flow-time is the only outcome-relevant factor identified in our study that can be modified. In this context we would to like to highlight the importance of a bundle of measures summarized as “systems saving lifes” [33].

## Limitations

Firstly, this study must consider limitations inherent to retrospective analysis of routine clinical data. Furthermore, this study collective does not reflect the whole cardiac arrest population, as we only analysed hospitalized non-traumatic OHCA cases. Documented prehospital chest imaging is rare (which would also cover non-hospitalized patients), therefore the real incidence of pneumothorax following cardiopulmonary resuscitation remains unknown. There is probably some degree of positive-selection in our study population. 38.0% of patients survived to hospital discharge in our study collective which is higher than previously reported in a large, multinational trial conducted in Europe (31.0%) [34]. Missing chest imaging led to exclusion, which occurs more often in patients who die immediately after admission. Due to the study design centred on a single physician-staffed ambulance, there is limited generalisability and a relatively small study population. No-flow-time was unknown in 30 cases.

There are some reports in the literature of a higher incidence of CPR-associated injuries when a mechanical CPR-device (m-CPR) is used [35, 36]. In our study population only 4 patients received m-CPR, a case number not suitable for further analysis.

We are aware that the study collective is heterogeneous regarding the chest imaging used. This explains why we could not assess additional chest injuries (e.g., rib or sternal fractures), which can be easily diagnosed by CT but not by sonography. Pneumothorax is a known complication of central venous catheter (CVC) placement, a procedure commonly performed in critically ill patients [37, 38]. We screened for cases where CVC insertion was performed prior to chest imaging that eventually found pneumothorax. We found 3 such cases, however, all of them had bilateral pneumothorax and unilateral attempts for CVC placement.

Finally, we acknowledge a “the chicken or the egg” problem. It is possible that pneumothorax cannot only be a result of CPR-induced trauma but also the underlying problem leading to cardiac arrest.

## Conclusions

In summary, pneumothorax after cardiopulmonary resuscitation is not an uncommon phenomenon. History of obstructive pulmonary disease seems to be a relevant risk factor contributing to the development of post-CPR pneumothorax.

## Data Availability

The datasets used and/or analysed during the current study are available from the corresponding author on reasonable request.

## Abbreviations

CPR: Cardiopulmonary resuscitation
OHCA: Out-of-hospital cardiac arrest
CPC: Cerebral performance category
CXR: Chest X-ray
CT: Computed tomography
ERC: European Resuscitation Council
EMS: Emergency medical service
ALS: Advanced Life Support
STROBE: Strengthening the Reporting of Observational studies in Epidemiology
AED: Automated external defibrillator
PESA: Pre Emergency Status Assessment
ROC: Receiver operating characteristics
AUC: Area under the curve
COPD: Chronic pulmonary obstructive disease
ECG: Electrocardiogram
CVC: Central venous catheter
